# The Impact of Diet and Gut Microbiota on Cognitive Health: A Comprehensive Review

**DOI:** 10.1002/alz70860_105330

**Published:** 2025-12-23

**Authors:** Samantha Ramachandra, Hamid R Sohrabi, Vincent Ho, BGDNK De Silva, Warnakulasuriya Mary Ann Dipika Binosha Fernando, Ralph N Martins

**Affiliations:** ^1^ Edith Cowan University, Perth, Western Australia, Australia; ^2^ Murdoch university, Murdoch, Western Australia, Australia; ^3^ Australian Alzheimer's Research Foundation, Perth, Western Australia, Australia; ^4^ Western Sydney University, Sydney, NSW, Australia; ^5^ University of Sri Jayewardenepura, Colombo, Western Province, Sri Lanka; ^6^ Alzheimer's Research Australia, Perth, Western Australia, Australia; ^7^ Alzheimer's Research Australia, Perth, Western Australia, Australia

## Abstract

**Background:**

The relationship between diet, gut microbiota, and neurocognition is pivotal in addressing age‐related cognitive decline and neurodegenerative diseases. Modifiable factors, particularly dietary patterns, significantly influence cognitive health and gut microbiota. Healthy diets like the Mediterranean (MeDi), DASH, and MIND promote microbial diversity, reduce neuroinflammation, and support cognitive function. In contrast, the Western Diet (WD), high in refined sugars and saturated fats, disrupts gut microbiota, lowers SCFA production, and exacerbates neuroinflammatory pathways, contributing to cognitive impairment.

**Method:**

A narrative review was conducted with the aim of summarising available scientific evidence to identify key dietary components responsible for the cognitive performances of adults and their effects on gut microbiota composition.

**Result:**

Macronutrients such as carbohydrates, proteins, fats, and micronutrients, including vitamins B, D, and E, and minerals like magnesium and zinc exhibit neuroprotective effects. Some nutrient components, such as omega‐3 fatty acids, reduce inflammation and oxidative stress in the brain and gut, while high dietary fiber enhances SCFA production and maintains gut barrier integrity. Notably, prebiotic and probiotic supplements have shown the potential to improve cognitive function by modulating gut microbiota composition. In clinical trials, probiotic strains such as Lactobacillus rhamnosus and Bifidobacterium longum have significantly improved cognitive levels and the level of anxiety.

Emerging evidence underscores the rapid and bidirectional response of gut microbiota to alteration of the dietary pattern. High‐fiber diets enhance beneficial bacteria, such as *Bifidobacterium* and *Akkermansia muciniphila*, while fiber‐deprived diets correlate with increased diversity of unhealthy bacteria, resulting in cognitive decline and hippocampal synaptic loss.

There is substantial evidence supporting the role of diet in mitigating cognitive decline and maintaining a healthy gut microbiota composition. This review highlights dietary components, such as plant‐based foods, probiotics, and prebiotics, which positively influence both cognitive health and gut microbiota balance. However, inconsistencies in findings from long‐term dietary intervention studies underscore the need for robust, large‐scale trials to establish causality.

**Conclusion:**

In conclusion, the review emphasizes the importance of personalized dietary strategies tailored to an individual's gut microbiota composition as a promising approach for preserving cognitive health and preventing neurodegenerative diseases, warranting further research in this area.

